# Pruritus in Systemic Diseases: A Review of Etiological Factors and New Treatment Modalities

**DOI:** 10.1155/2015/803752

**Published:** 2015-07-09

**Authors:** Nagihan Tarikci, Emek Kocatürk, Şule Güngör, Ilteriş Oğuz Topal, Pelin Ülkümen Can, Ralfi Singer

**Affiliations:** Department of Dermatology, Okmeydanı Training and Research Hospital, 34384 Istanbul, Turkey

## Abstract

Pruritus is the most frequently described symptom in dermatology and can significantly impair the patient's quality of life. In 10–50% of adults with persistent pruritus, it can be an important dermatologic clue for the presence of a significant underlying systemic disease such as renal insufficiency, cholestasis, hematologic disorder, or malignancy (Etter and Myers, 2002; Zirwas and Seraly, 2001). This review describes the presence of pruritus in different systemic diseases. It is quite important to discover the cause of pruritus for providing relief for the patients experiencing substantial morbidity caused by this condition.

## 1. Pruritus

Pruritus is a topic that has caused a great deal of controversy because it is difficult to characterize and define. Various indirect definitions proposed include a sensation which provokes the desire to scratch or an uneasy sensation of irritation in the skin [1].

**Figure 1:**
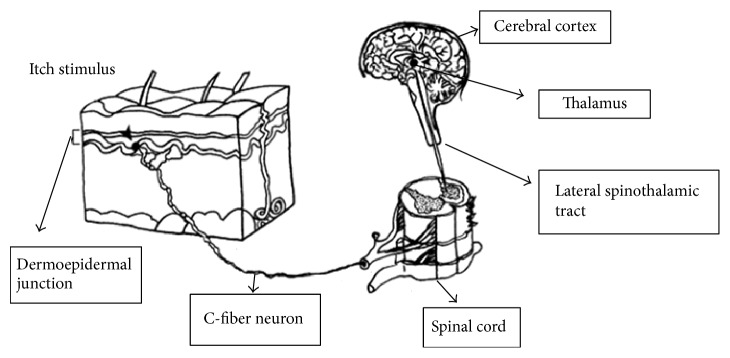
Neurological pathways of pruritus.

The itch impulse is transmitted from peripheral nerves to the dorsal horn of the spinal cord, across the cord via the anterior commissure, and ascendingly along the spinothalamic tract to the laminar nuclei of the contralateral thalamus. Thalamocortical tracts of tertiary neurons are believed to relay the impulse through the integrating reticular activating system of the thalamus to several areas of the cerebral cortex. Pruritus may be caused by some chemical substances as histamine, prostaglandins, proteases, and substance P (Figure 1) [2, 3]. This review describes the existence of pruritus in different internal disorders. Systemic causes of pruritus are as follows.

*Metabolic Disorders*. Chronic renal failure (dialysis) and liver diseases with or without cholestasis. 

*Infections*. HIV, hepatitis C virus. 

*Hematologic Diseases*. Iron deficiency, polycythemia vera. 

*Endocrinal Disorders*. Thyroid diseases, diabetes mellitus. 

*Paraneoplastic Diseases*. Lymphomas and solid organ tumors.

## 2. Uremic Pruritus

Of all the systemic diseases associated with pruritus, renal impairment is probably the most common underlying pathology. In older series, up to 90% of patients were afflicted with pruritus, but now between 20% and 50% are affected [4].

Uremic pruritus (UP) is one of the frequent complications in terminal renal disease patients and it is not present in acute renal failure. Pruritus affects 50–90% of patients undergoing peritoneal dialysis or hemodialysis; symptoms usually begin about six months after the start of dialysis and range from localized and mild to generalized and severe [5].

The mechanism underlying uremic pruritus seems to depend on many factors, including dryness of the skin, secondary hyperparathyroidism, divalent ion abnormalities, hypervitaminosis A, peripheral neuropathy and neurological changes, inflammation, abnormal mast-cell proliferation in skin of patients on hemodialysis, and elevated levels of serotonin concentrations or some combination of these. However, present data point toward a central role of immune and opioidergic systems [6].

A new hypothesis of glycation with advanced glycation end products (AGES) accumulation in stratum corneum has been proposed as a possible underlying cause of UP [7]. In 25% of patients with UP, pruritus is most severe during or immediately after dialysis, probably due to antigen sensitization from dialysis membranes. And a recent noncontrolled study showed the use of polymethylmethacrylate high-flux dialysis membranes to be associated with a significant reduction in pruritus scores [8].

Xerosis, or dry skin, is the most frequent dermatological manifestation in patients undergoing dialysis therapy. Morton et al. [9] assessed the prevalence and severity of pruritus and skin dryness in a uremic population receiving maintenance dialysis and demonstrated that pruritic skin of patients undergoing hemodialysis or peritoneal dialysis had significantly lower hydration than dialysis patients without uremic pruritus.

However Yosipovitch et al. [10] and Ståhle-Bäckdahl [11] did not find a correlation between the severity of pruritus and objective parameters of skin dryness. It was hypothesized that uremic xerosis, even if it is not the primary cause of pruritus, has a worsening effect by reducing the threshold for itch [12].

The clinical characteristics of End Stage Renal Disease (ESRD) pruritus are variable. In two-thirds of patients, pruritus is generalized, while in the remaining patients it predominantly affected the back, the face, and arteriovenous fistula arm, in this order of frequency. Pruritus may be constant or intermittent and it is usually worst at night [13]. Uremia causes severe paroxysms of pruritus, especially during the summer, and some patients report pruritus during or soon after dialysis [4].

Despite the evidence for release of histamine, there was no correlation found between plasma histamine levels and severity of pruritus and antihistamines lack any activity in uremic patients suggesting that plasma histamine does not play a remarkable role in uremic pruritus [14].

Correcting anemia with erythropoietin, pruritus improved within one week, probably due to reduction in plasma histamine concentrations as a result of decreased production of histamine-releasing cytokines [15].

Secondary hyperparathyroidism is another common problem in patients on dialysis. Secondary hyperparathyroidism leads to microprecipitation of calcium and magnesium salts in the skin. That causes degranulation of mast cells and release of serotonin and histamine. Dramatic relief of pruritus after subtotal parathyroidectomy has been reported [16]. It has been reported that pruritus can exactly disappear after parathyroidectomy. On the other hand, not all patients with severe hyperparathyroidism have pruritus [17].

Once chronic pruritus has occurred, there may be secondary changes in nerves in the skin and possibly the central nervous system which heighten the perception/sensation of itch (central sensitization) [4]. Jedras et al. [18] found nervous dysfunction especially the somatic component related to pruritus in uremic patients. On the other hand, reports showing the efficacy of lidocaine, capsaicin, and gabapentin in controlling uremic pruritus are in favor of a relationship between neuropathy and itching in HD patients.

Capsaicin, a natural alkaloid found in the chili pepper plant, also reduces levels of substance P in cutaneous type C sensory nerve endings and is significantly alleviated in uremic pruritus patients [19]. Recent studies showed that vanilloid receptors on cutaneous sensory nerve fibers are potential targets for antipruritic therapy and they have important role in itch. Vanilloid receptor subtype 1 (TRPV1) ligand was originally described to be activated by capsaicin [20]. Peripheral neuropathy may affect the perception of pruritus. This, in turn, may explain the apparent efficacy of lidocaine in uremic pruritus. However, this drug may be too toxic in uremic patients.

For therapy general measures include a cool environment, loose clothes, and the frequent use of emollients such as aqueous cream [21]. Local treatment by topical tacrolimus 0.03% ointment twice daily has recently been advocated for short-term management of uremic pruritus by Kuypers et al. [22]. A cream containing high concentration of gamma linolenic acid (GLA), an essential fatty acid derived from certain plant seed oils, was tested on pruritic uremic patients and so it was suggested that GLA can exert an improved antipruritic effect [23].

Phototherapy with UVB, particularly broadband UVB (wavelength 280–315 nm), has been used for more than a decade for ESRD-associated itch and is still considered a treatment of choice in many centers. NB-UVB and UVA therapy is not effective alone [24]. It is important to inform the patient that the antipruritic effect is noticed only after 1-2 months of treatment and it can aggravate itching in the first 2 weeks [25]. The possible antipruritic mechanism of uvb is to decrease proinflammatory cytokines. Starting at three times per week is sufficient to induce remission and ongoing treatment once or twice weekly can often control the pruritus [26].

Uremic pruritus is partly a result of an imbalance in the opioidergic system, with hyperactivity of *μ*-opioid receptors in dermal cells and lymphocytes [27]. Endogenous opioids are partially excreted by the kidney and serum beta endorphin levels are elevated in chronic renal failure. Opioid peptides cause pruritus by degranulation of cutaneous mast cells, or through a direct central and peripheral pruritogenic effect by activating *μ*-opioid receptors.

Naltrexone, oral *μ*-opioid antagonist, showed dramatic response in the treatment of renal pruritus. Recently, nalfurafine (TRK-820), a kappa-receptor agonist, has also shown beneficial effects in the severe ESRD-associated pruritus. A disadvantage of nalfurafine is that it is only available in an intravenous formulation [28].

Endocannabinoid system may contribute to the pathophysiology of itch. But its role in the modulation of chronic itch in systemic diseases has not been investigated yet. Mirtazapine is effective in uremic pruritus which is an antidepressant, presynaptic alpha2 adrenergic inhibitor, and a potent antagonist of serotonin and histamine receptors. The antianxiety property of mirtazapine may indirectly reduce itch [29, 30].

In a study, 100–300 mg of oral gabapentin administrated after each hemodialysis session was an effective and safe regimen for ESRD pruritus. It was recommended to start with a lower dose of gabapentin with slow upward titration to avoid the risk of gabapentin-induced neurotoxicity and coma in ESRD patients. Other reported adverse effects of gabapentin include fatigue and nausea [31].

Thalidomide used as an immunomodulatory agent suppresses TNF alfa production and can be effective in the treatment of ESRD-associated pruritus. It was speculated that the antipruritic action of it may result from a central depressant effect. Its use is limited by significant adverse effects: drowsiness, birth defects, and irreversible peripheral neuropathy [32].

Oral use of activated charcoal has been shown to completely resolve or significantly reduce pruritus symptoms in patients on chronic dialysis [33].

A Japanese study and the DOPPS (Dialysis Outcomes and Practice Patterns Study) demonstrated an association between UP and an increased risk of mortality and successful renal transplantation is the only definitive treatment for the pruritus of chronic renal failure [13].

## 3. Hepatogenic Pruritus

Pruritus is a common symptom in patients with liver disease and cholestasis. It occurs in approximately 20–25% of jaundiced patients [34].

It is more common in intrahepatic than extrahepatic cholestasis. Intrahepatic cholestatic itch is usually associated with chronic viral hepatitis, cholestasis of pregnancy, primary biliary cirrhosis, and Alagille syndrome which is a pediatric cholestatic syndrome. Extrahepatic cholestatic itch may be caused by pancreatic tumor or pseudocyst, pressure on the bile ducts due to a nearby mass or tumor, and primary sclerosing cholangitis (PBC) [25, 35].

Approximately 80% of patients with PBC complain from itching and in 50% of patients it is the presenting symptom. This may suggest pruritus as being a potential clinical marker for PBC, aiding in early diagnosis [35].

Cholestatic pruritus with or without liver injury complicates the use of oral contraceptives, phenothiazines, tolbutamide, anabolic steroids, and other drugs. It can occur after weeks to months from the start of treatment [36].

Pruritus in hepatic disease can be severe leading to sleep deprivation and have a marked negative impact on quality of life; when persistent, it is an indication for liver transplantation even in the absence of hepatic insufficiency [37].

The unique feature of cholestatic pruritus is most severe at night, with a predilection for the hands and feet as well as areas where clothes are rubbing, but itch may also be generalized. The intensity of pruritus undergoes a circadian rhythm. It is often generalized and described with terms such as “lying on a bed of cactus,” “pins and needles,” and “crawling” by patients and unlike other causes of pruritus, scratching does not appear to relieve cholestatic pruritus [38, 39].

In recent years several mechanisms are generally accepted as possible explanations to the pathophysiology of pruritus in cholestatic liver disease. It is suggested that cholestasis leads to release of toxic pruritogens from the liver; this stimulates neural itch fibers in the skin, which transmit the stimulus to the spinal cord and afterwards the brain [40].

The pruritogens in cholestasis are not yet defined, although bile salts, bile acids, bilirubin progesterone metabolites, histamine, and endogenous opioids accumulating in circulation and tissues have been historically considered as major causes for cholestatic pruritus [41]. However, it is clear that in many cholestasis patients bile acid resins do not improve itch and bile acid levels in skin and serum or serum markers of liver disease do not show a reliable correlation with degree of pruritus [42].

Over the past decade, pruritus of cholestasis was thought to be centrally mediated by endogenous opioids but as yet there is no completely defined role in pruritus of cholestasis [38]. The *μ*-opioid and kappa opioid receptors may act as modulators of itch in the central nervous system of animals. The *μ*-opioid receptor agonists are pruritogens, while kappa receptor agonists are antipruritic [43].

Elevated serum levels of endogenous opioids were observed in the plasma of cholestatic PBC patients. Opioids are thought to cause pruritus by modifying the sensation of itch both centrally and peripherally [44].

The *μ*-opioid antagonists, such as naltrexone and naloxone, have been proven to be efficient in the treatment of cholestatic pruritus [45]. As naloxone has a short duration of action and only an intravenous dosing route, it is not practical for the treatment of pruritus. Naltrexone has an oral-dosing route and so can be used as an effective antipruritic, especially in this kind of pruritus. The main side effects are nausea, vomiting, fatigue, and dizziness [46].

Recent studies suggest that autotaxin (ATX), the enzyme that converts lysophosphatidylcholine into lysophosphatidic acid (LPA), is a potential mediator of cholestatic pruritus [47]. The increased local formation of LPA near unmyelinated nerve endings potentiates action potential along the nerve fibers and correlates with the itch response [39, 48]. Serum ATX activity is especially increased in patients with cholestatic pruritus and closely correlates with the effectiveness of therapeutic interventions [49] Consequently, autotaxin may play an important role as a potential target in the treatment of pruritus in patients with cholestatic liver disease [47].

The mechanism of the reported antipruritic effect of rifampicin which is a P450 cytochrome enzyme inducer may be explained, at least partly, by the Pregnane X receptor- (PXR-) dependent transcriptional inhibition of ATX expression. Rifampicin can be hepatotoxic and liver tests should be monitored. Thus, ATX likely represents a novel therapeutic target for pruritus of cholestasis [49].

Several studies indicated that females with cholestatic pruritus usually reported worse itch premenstrually, during hormone replacement therapy, and, in 0.5% of pregnant women, particularly during the third trimester [50]. Pruritus gravidarum usually resolves soon after delivery but may develop with subsequent pregnancies or with oral contraceptive ingestion [51]. Pruritus in intrahepatic cholestasis of pregnancy is characterized by pruritus and elevated levels of bile acids, and it carries a high risk of adverse perinatal outcome [39].

With regard to chronic cholestatic hepatic disease, plasma histamine levels are higher in patients with pruritus than without pruritus. However, antihistamines are mostly ineffective in cholestatic pruritus [52]. Any beneficial effect may be due to their sedative properties [53].

Cholestyramine, colestipol, and colesevelam are nonabsorbable anion exchange resins approved to treat hypercholesterolemia. The idea behind the administration of these resins to treat the pruritus of cholestasis is to enhance the intestinal excretion of the pruritogens [54].

Hepatic enzyme inducers, such as phenobarbital and flumecino, are used to manage patients with the pruritus of cholestasis. The reported decrease in pruritus associated with phenobarbital may be caused by sedation [55, 56].

Intravenous 5-HT3-receptor antagonist ondansetron has been shown to have antipruritic effect in cholestatic patients with conflicting results [57]. It may also have an effect on opioid pathways. Interestingly, the serotonin reuptake inhibitor sertraline was shown to moderately improve pruritus in cholestatic patients [58].

Anecdotal reports of successful treatment of cholestatic pruritus include the use of intravenous lidocaine, UVB phototherapy, and androgens [21, 59].

## 4. Paraneoplastic Pruritus

Paraneoplastic pruritus is defined as itch that occurs early during the course or even precedes the clinical evidence of the malignancy. It is not caused by the pressure or invasion of the neoplastic mass and relieves after the removal of the tumor. Although it is considered to be a common phenomenon, malignant disease can be detected in only less than 10% of patients with chronic itch. Lymphoma and leukemia were the most common malignancies [60]. In persistent and widespread itching; hodgkin's lymphoma, leukemia, polycythemia vera and multiple myeloma should be considered [61].

Paraneoplastic pruritus has occurred with such high frequency that some researchers have even proposed that itch should be considered a B symptom of Hodgkin's disease. Itch has been known for decades to be the most common symptom of Hodgkin's lymphoma (HL) and it occurs in about 30% of patients. Generalized pruritus often occurs months or even a year before Hodgkin disease is first diagnosed. It may be an important clue for the detection of occult Hodgkin's disease in previously healthy patient [62].

In several cases, paroxysms of generalized itching and hyperhidrosis have been observed and itch can present with ichthyosiform skin changes on the extremities, prurigo nodularis, or as a new onset of eczema lesions with Hodgkin's disease [60, 63]. It is often worse at night and starts in the legs and later engages the whole body. Generalized pruritus tends to occur more often in the nodular sclerosis type of Hodgkin's disease with mediastinal involvement [64].

More recent reports suggest that patients with hodgkin's lymphoma and mycosis fungoides suffering from severe pruritus have lower response to treatment than stage matched nonpruritic patients. Itching in hodgkin's lymphoma patients, may be a prognostic significance, as these patients often experience more aggressive disease [65].

Pruritus in Hodgkin's disease is thought to be caused by release of histamine, since it responds to histamine blockers like cimetidine. Eosinophilia associated with the pleomorphic infiltrate of HL and high serum levels of IgE may be contributing factors to histamine release and the pathogenesis of pruritus in HL. But pruritus may follow cholestasis and disturbed central neurotransmission, because of its beneficial response to mirtazapine. Another proposed mechanism of itch in HL is the release of pruritogens such as leukocyte peptidases and bradykinins due to an autoimmune response to lymphoid cells [66].

Pruritus is an uncommon presentation of chronic leukemia, myelomatosis, and lymphosarcoma. It is more often in lymphatic than in granulocytic forms [64].

Pruritus can also be a paraneoplastic sign in solid tumors including lung, colon, brain, and breast and gastric tumors, adenocarcinoma, or squamous cell carcinomas of different organs such as the prostate and laryngeal tumors. In patients with extrahepatic cholestasis, itch may be caused by obstructive tumor in the pancreatic head and primary sclerosing cholangitis. Intractable paraneoplastic pruritus has been reported as an initial presentation of insulinoma [61, 67].

## 5. Haematological Pruritus

*Iron deficiency,* with or without anemia, is often regarded as a cause of generalized pruritus and signs of iron deficiency in addition to pruritus include glossitis and angular cheilitis [68].

In men, iron deficiency is mostly related to alcoholism or cancer. Generalized pruritus with iron deficiency, particularly in the elderly male, is a reason for alert, suggesting the obligation to include serum ferritin, iron fecal occult blood, and urinalysis to be done as soon as possible. The iron is necessary for activity of many enzymes. Alterations in their function may lead to the metabolic disturbances and itching [53].

Pruritus due to iron deficiency responds to iron supplementation, which should be continued for 3 months after haemoglobin levels are back to normal [68].

*Polycythemia vera* is a myeloproliferative disorder characterized by erythrocytosis that leads to an elevated haemoglobin and erythrocyte mass [69]. Itch is experienced by 30–50% of patients with polycythaemia rubra vera. Pruritus is characteristically precipitated usually by contact with water during bath or shower at any temperature, but less frequently with cold water. It is important to note that contact water is not the only way in which itching is triggered in patients with PV but sweating after exercise, alcohol consumption, and sudden change in temperature may also result in pruritus [70, 71].

Pruritus is a particular feature of PV which may precede diagnosis by several years. Itching may be so severe that patients refuse to bathe and many patients believe that it is the most troublesome aspect of PV [70, 72].

In a study proximal extensor surfaces of limbs, interscapular area, chest and abdominal wall are the most described distributions of this condition [73]. It therefore seems likely that in PV patients a different cutaneous distribution of mast cells may occur, which causes a more pronounced proximal pruritus [74].

Due to the observation that aspirin alleviates this particular form of pruritus, the impressive response to paroxetine supports the role of platelet, serotonin, and prostaglandins in polycythaemia vera associated pruritus. However, the concentration of platelet serotonin was similar in patients with PV with and without pruritus and no functional abnormalities of platelets were found [72].

A study showed that in patients with PV the basophilic granulocytes are constitutively activated and are hypersensitive and play a key role in triggering symptoms [75].

Recommended strategies in PV are as follows: alkalinization of bathing water with sodium bicarbonate, topical capsaicin treatment, systemic therapy with antihistaminics and antiserotonergic drugs, or phototherapy [70].

Cyproheptadine and pizotifen were effective in decreasing pruritus; they are also strong antagonists of serotonin and histamine [76].

Cytoreductive therapy with agents such as hydroxyurea and interferon alfa has been noted to be effective in the control of pruritus associated with PV [77].

## 6. Endocrine Disorders

Pruritus occurs in 4–11% of patients with thyrotoxicosis, particularly with long-lasting, untreated Graves' disease. Increased blood flow and skin temperature and decreased itch threshold are a hypothesis on how excess of thyroid hormones may lead to itch. Hyperthyroidism associated pruritus may also occur as a result of cholestatic jaundice in some cases [78].

Myxoedema and hypothyroidism associated pruritus is rare and may be related to the dryness of skin which is seen in 80–90% of patients. This itching responds to emollients and thyroid hormone replacement [79].

Abnormal parathyroid gland activity usually occurs in the context of chronic renal failure that may also cause pruritus [80]. Although no correlation between itch and serum parathyroid hormone levels has ever been found, subtotal parathyroidectomy may result in a dramatic resolution of pruritus for some patients. However, this result may not be sustained and surgery certainly does not work for all patients [17].

In diabetes mellitus, generalized pruritus is probably very rare but localized pruritus in the perianal/genital region occurs in diabetic women more frequently which is due to* Candida albicans* or dermatophyte infection and it is not clear whether metabolic abnormalities due to renal failure, autonomic dysfunction with anhidrosis, or diabetic neuropathy are responsible for this phenomenon [81].

It is suggested that hormonal deficit in women in the postmenopausal period may provoke vulvar pruritus [82].

## 7. HIV and HCV Infections

Pruritus is one of the most common symptoms encountered in HIV infection and can even be the initial presentation so it may be important in early diagnosis. Pruritus in HIV may occur with skin infections, infestations, papulosquamous disorders, photodermatitis, xerosis, drug reactions, and occasionally lymphoproliferative disorders such as cutaneous T cell lymphoma or without primary dermatosis [83].

Pruritus accompanied by hypereosinophilia may be used to define a subset of HIV-seropositive individuals showing prototypic hyperactivation of humoral immunity; these patients have a high HIV viral load [84].

A possible correlation was observed between intractable resistant pruritus and augmented HIV viral load. The presence of pruritus should stimulate more in-depth analyses and more aggressive therapeutic approach [84].

Pruritus has been reported in up to 15% of patients with chronic HCV infection and may be a presenting symptom. The pathogenesis of HCV-related itch is still obscure. In the absence of cholestasis, itch may be an adverse effect of antiviral therapy, as it happens in up to 29% of patients treated with interferon alfa plus ribavirin [85].

## 8. Conclusion

Systemic causes must be considered, especially in elderly patients in whom pruritus is persistent and refractory to xerosis management and other nonspecific therapies. Therefore, a thorough history and physical examination is essential in the evaluation of chronic pruritus [86].

A multidisciplinary approach with a dermatologist, psychiatrist, and internist is required to prevent psychiatric morbidity and possible deterioration of quality of life in these patients. Also one of the most important tasks for the dermatologist is to thoroughly explain to the patient to keep the skin hydrated and avoid skin drying activities such as hot bathing, dry environment, using alkali soap, and wearing irritating fabric.
